# Supplementary figures and images for: Bladder Microbiota Are Associated with Clinical Conditions That Extend beyond the Urinary Tract
Source: Microorganisms. 2022 Apr 22;10(5):874. doi: 10.3390/microorganisms10050874 (PMC9147640; doi:10.3390/microorganisms10050874)

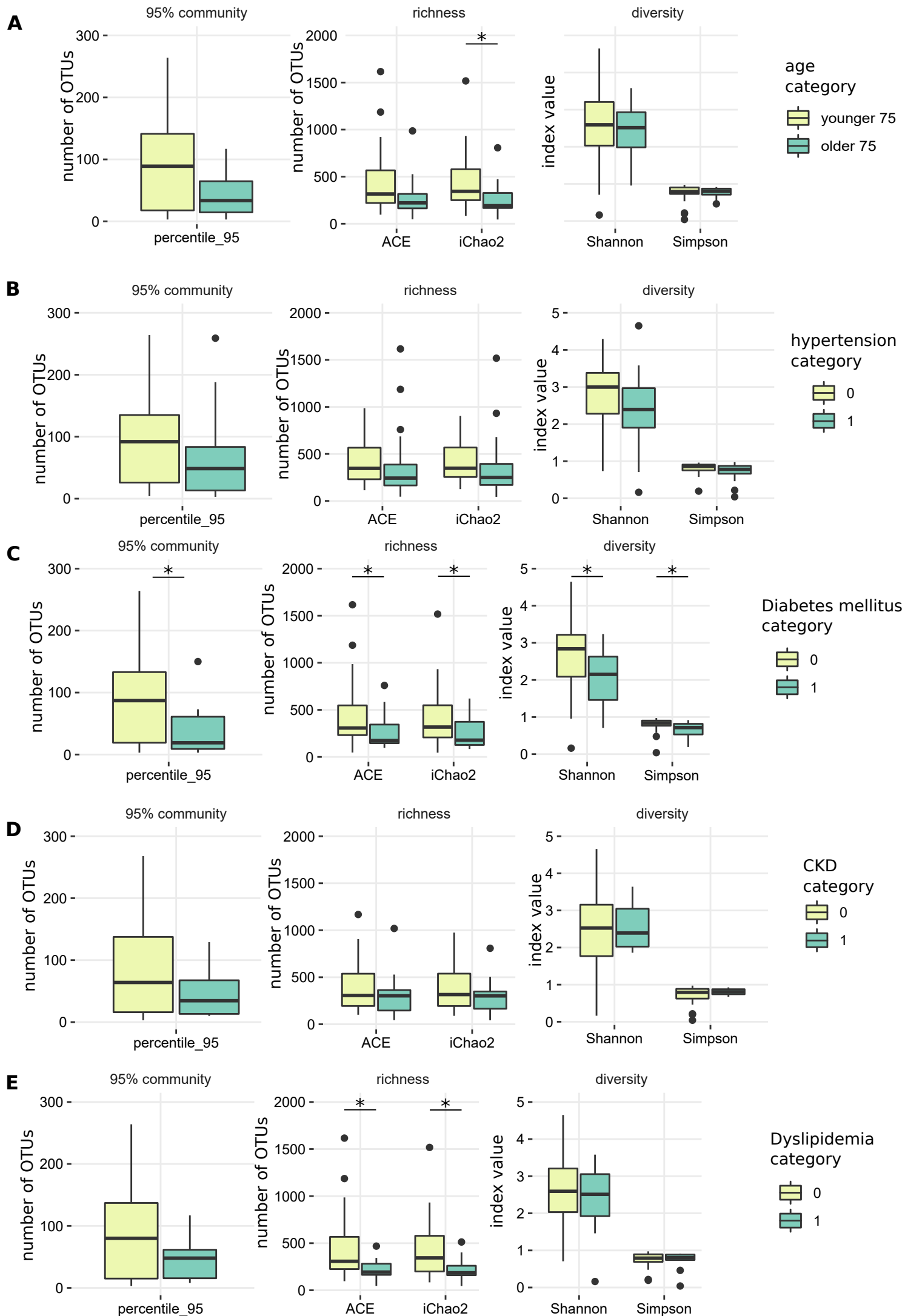

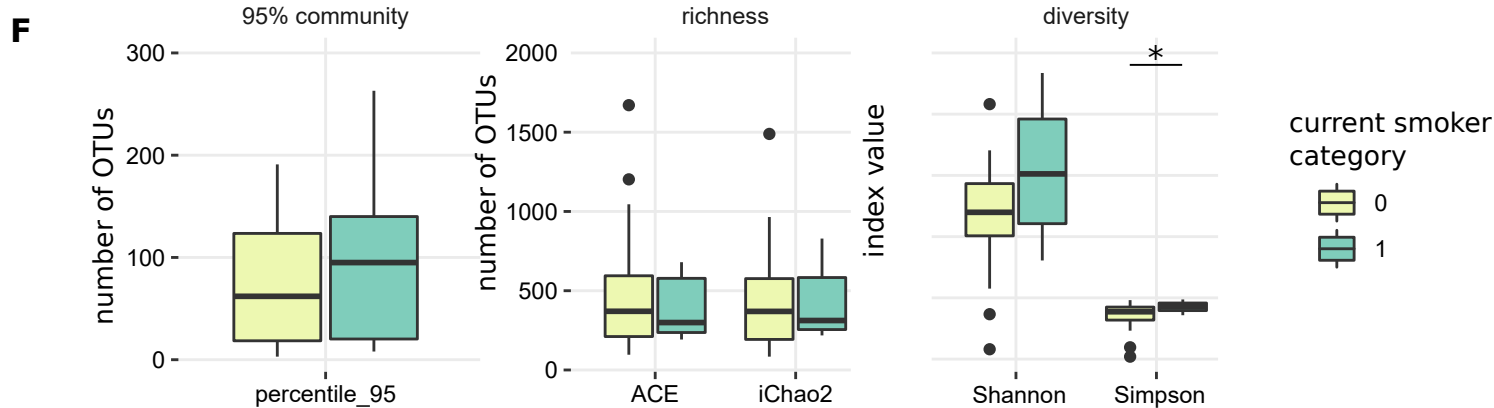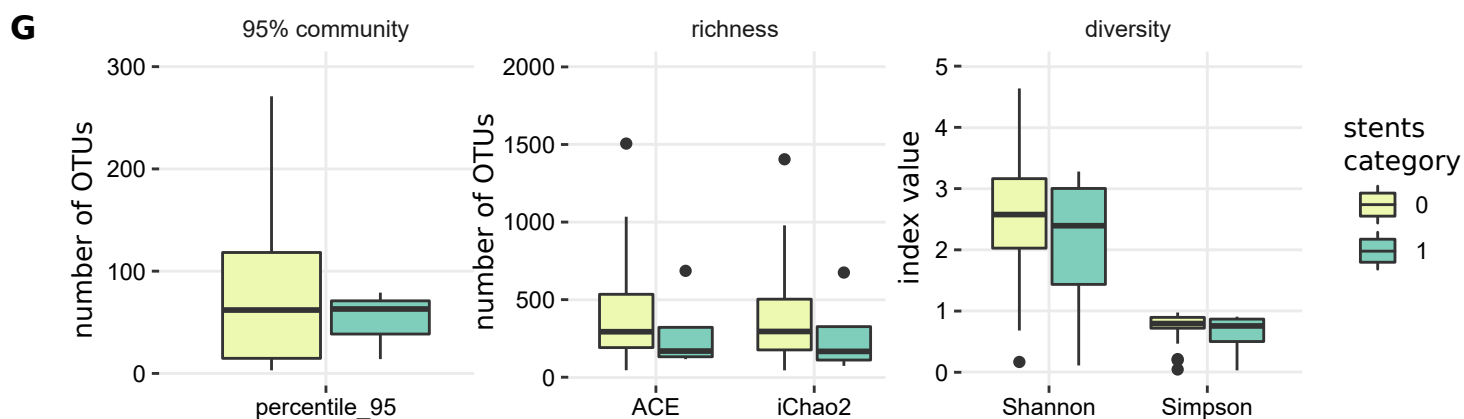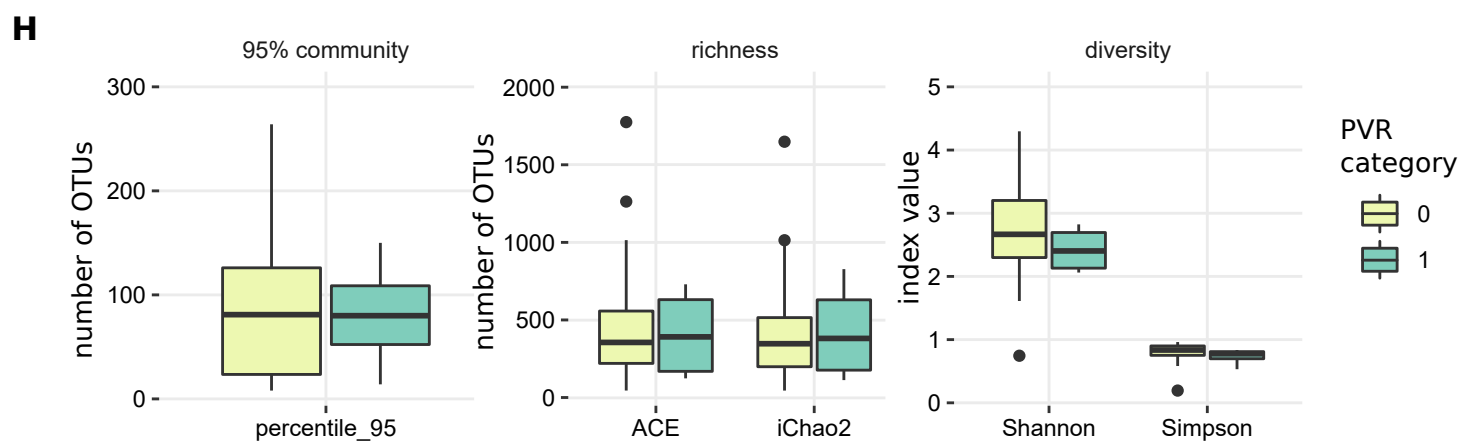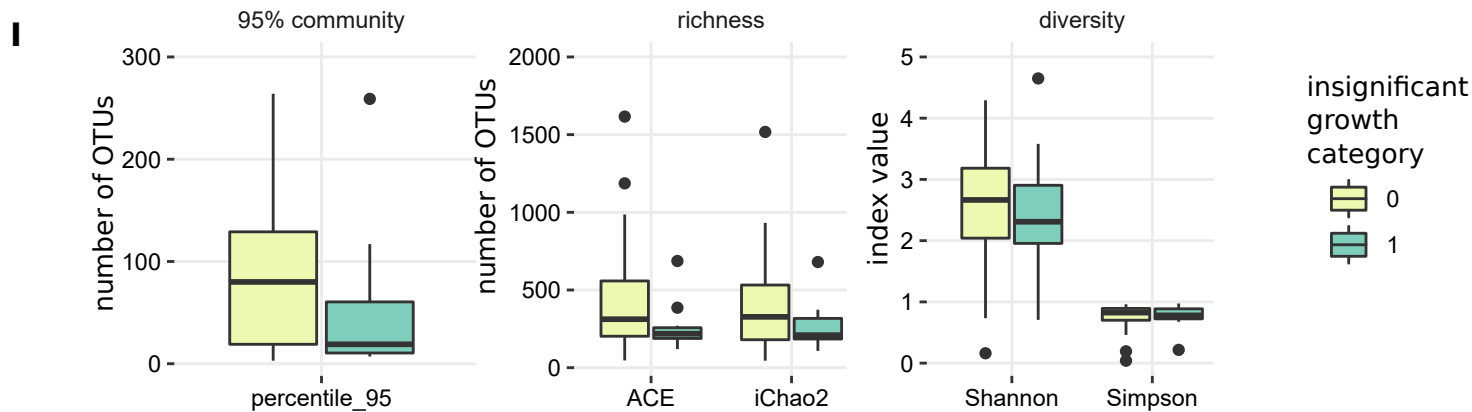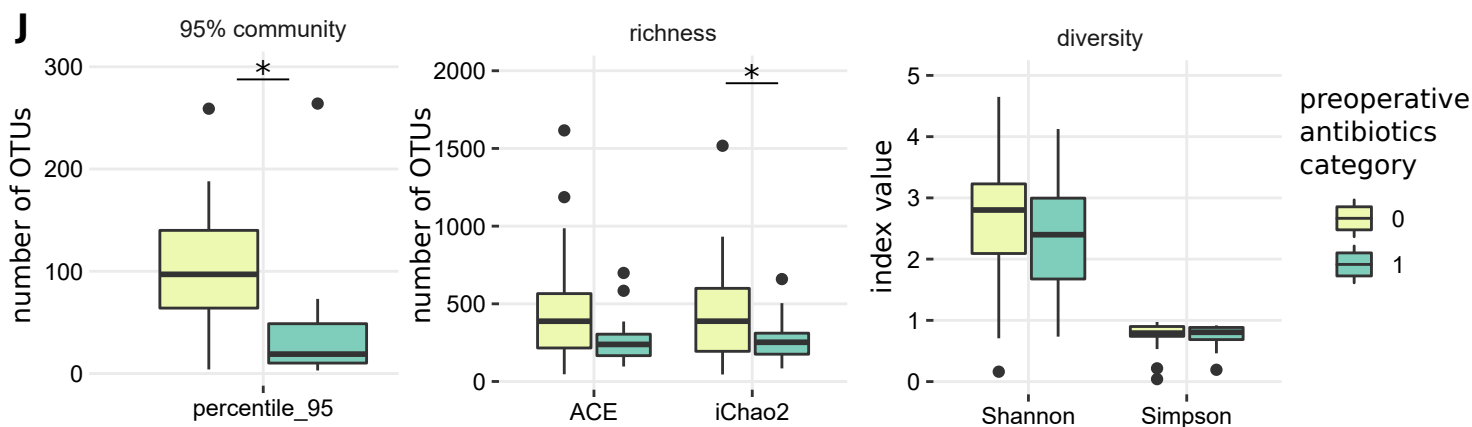

Supplement: Supplementary file 1 [file microorganisms-10-00874-s001.zip › Supplementary_figure_S1.pdf]
